# Supplementary material for: BIRC6 modifies risk of invasive bacterial infection in Kenyan children
Source: eLife. 2022 Jul 22;11:e77461. doi: 10.7554/eLife.77461 (PMC9391038; doi:10.7554/eLife.77461)
Supplement: Transparent reporting form [file elife-77461-transrepform1.docx]

***eLife’s* transparent reporting form**

We encourage authors to provide detailed information *within their submission* to facilitate the interpretation and replication of experiments. Authors can upload supporting documentation to indicate the use of appropriate reporting guidelines for health-related research (see [EQUATOR Network](http://www.equator-network.org/%20)), life science research (see the [BioSharing Information Resource](https://biosharing.org/)), or the [ARRIVE guidelines](http://www.plosbiology.org/article/info:doi/10.1371/journal.pbio.1000412) for reporting work involving animal research. Where applicable, authors should refer to any relevant reporting standards documents in this form.

If you have any questions, please consult our Journal Policies and/or contact us: [editorial@elifesciences.org](mailto:editorial@elifesciences.org).

**Sample-size estimation**

- You should state whether an appropriate sample size was computed when the study was being designed
- You should state the statistical method of sample size computation and any required assumptions
- If no explicit power analysis was used, you should describe how you decided what sample (replicate) size (number) to use

Please outline where this information can be found within the submission (e.g., sections or figure legends), or explain why this information doesn’t apply to your submission:

The sample size for the study was pragmatic, with all paediatric cases of bacteraemia and severe malaria presenting to Kilifi County Hospital during the study period being included. Recruitment to the study is described in “Study samples” in the Materials & Methods section (lines 352-366).

**Replicates**

- You should report how often each experiment was performed
- You should include a definition of biological versus technical replication
- The data obtained should be provided and sufficient information should be provided to indicate the number of independent biological and/or technical replicates
- If you encountered any outliers, you should describe how these were handled
- Criteria for exclusion/inclusion of data should be clearly stated
- High-throughput sequence data should be uploaded before submission, with a private link for reviewers provided (these are available from both GEO and ArrayExpress)

Please outline where this information can be found within the submission (e.g., sections or figure legends), or explain why this information doesn’t apply to your submission:

Sample (lines 394-398 & 406-409) and SNP (lines 388-393 & 412-417) exclusion criteria are detailed in “Genotyping & imputation” in the Materials & Methods section.

The study uses genotyping data generated by the Wellcome Trust Case-Control Consortium 2 (bacteraemia samples and controls) and the MalariaGEN consortium (severe malaria samples and controls). Genotype and phenotype data for the study samples are available via the European Genome-Phenome Archive, with accession codes EGAD00010000950 and EGAD00010000904.

**Statistical reporting**

- Statistical analysis methods should be described and justified
- Raw data should be presented in figures whenever informative to do so (typically when N per group is less than 10)
- For each experiment, you should identify the statistical tests used, exact values of N, definitions of center, methods of multiple test correction, and dispersion and precision measures (e.g., mean, median, SD, SEM, confidence intervals; and, for the major substantive results, a measure of effect size (e.g., Pearson's r, Cohen's d)
- Report exact p-values wherever possible alongside the summary statistics and 95% confidence intervals. These should be reported for all key questions and not only when the p-value is less than 0.05.

Please outline where this information can be found within the submission (e.g., sections or figure legends), or explain why this information doesn’t apply to your submission:

For statistical methods please see “Association analysis & fine mapping”, “Replication samples & analysis”, “Bayesian comparison of models of association” and “Co-localisation analysis”. Exact p-values and Ns are reported throughout.

(For large datasets, or papers with a very large number of statistical tests, you may upload a single table file with tests, Ns, etc., with reference to sections in the manuscript.)

**Group allocation**

- Indicate how samples were allocated into experimental groups (in the case of clinical studies, please specify allocation to treatment method); if randomization was used, please also state if restricted randomization was applied
- Indicate if masking was used during group allocation, data collection and/or data analysis

Please outline where this information can be found within the submission (e.g., sections or figure legends), or explain why this information doesn’t apply to your submission:

For details of case and control recruitment see “Study samples” in the Materials & Methods section (lines 352-366).

**Additional data files (“source data”)**

- We encourage you to upload relevant additional data files, such as numerical data that are represented as a graph in a figure, or as a summary table
- Where provided, these should be in the most useful format, and they can be uploaded as “Source data” files linked to a main figure or table
- Include model definition files including the full list of parameters used
- Include code used for data analysis (e.g., R, MatLab)
- Avoid stating that data files are “available upon request”

Please indicate the figures or tables for which source data files have been provided:

Patient level genotype and phenotype data are available via the European Genome-Phenome Archive, with accession codes EGAD00010000950 (WTCCC2: bacteraemia cases and controls) and EGAD00010000904 (MalariaGEN Consortium: severe malaria cases and controls). Full GWAS summary statistics have been deposited with the GWAS Catalog with accession code GCST90094632.

Code and source data underlying each figure are available at: <https://github.com/jjgilchrist/Kenya_bacteraemia_malaria>
